# Conjoined Twins

**Published:** 2018-06-30

**Authors:** Pramod Kattel

**Affiliations:** 1 Department of Obstetrics and Gynaecology, Kathmandu National Medical College, Kathmandu, Nepal

**Keywords:** *conjoined twins*, *monozygotic twins*, *Siamese twins*, *twinning*, *twin pregnancy*

## Abstract

Reported here is a case of conjoined twins presented to ante-natal outpatient department of Paropakar Maternity and Women's Hospital on 21^st^ August 2015 on a 20 year "Primigravida at 27 weeks and 6days of gestation not in labor" referred from a polyclinic following ultrasonography diagnosis for better management. After confirming the diagnosis and counseling the patients regarding mode of delivery and possible outcomes, elective caesarean section was performed and delivered male conjoined twins of Parapagusdicephalus type with poor Apgar score. No resuscitation attempted except oxygen supplementation as per wish of parents and early neonatal deaths occurred at one hour of life.

## INTRODUCTION

Conjoined twins are rare anomalous presentation among symmetric monozygotic twin pregnancy. Their incidence is about 1 in 60,000 pregnancies.^1,2^ They are of same sex and karyotype. They are more common in females with male to female ratio being 1:3.^3^ Their exact etiology are not known. They result from incomplete splitting of an embryo into two separate twins after 12 days of conception.^1,4^

## CASE REPORT

Mrs P. Tamang, 20 year Primigravida presented to antenatal checkup (ANC) out-patient department (OPD) of Paropakar Maternity and Women's Hospital (PMWH) on 21^st^ August 2015 as first visit at 27 weeks and 6 days of gestation by last menstrual period with obstetric scan done at a polyclinic on 19^th^ August 2015 showing viable conjoined twin of Parapagus type.

On examination, her general condition was good with no fresh complains and perceiving good fetal movement. Her general examinations were normal with stable vitals and normal systemic examination. Abdominal examination revealed 34 week sized uterus in longitudinal lie with palpable two heads at lower pole.

Then repeat obstetric scan was performed on same day at PMWH which showed viable conjoined twin in cephalic presentation of 24^+^ weeks with anterior placentation and normal liquor. Then elective caesarean section (CS) was planned for 23^rd^ August 2015. Patient was sent back after sending necessary blood investigations for CS and advised to come at emergency department on 23^rd^ August 2015 on nothing per oral status from 10:00 pm of 22^nd^ August. Following patients arrival at emergency department with reports on 23^rd^ August, patient was taken to operation theatre with diagnosis of "Primi at 28 weeks and 1 day of gestation with conjoined twins not in labor" and CS was performed with J-shaped uterine incision on left side and baby delivered by vertex with following operative findings.

Lower uterine segment- not well formed; liquor- clear and adequate; presentation- cephalic; placenta- anterior (monochorionic, monoamniotic); bilateral tubes and ovaries -normal and estimated blood loss- 300ml.

**Figure 1.**
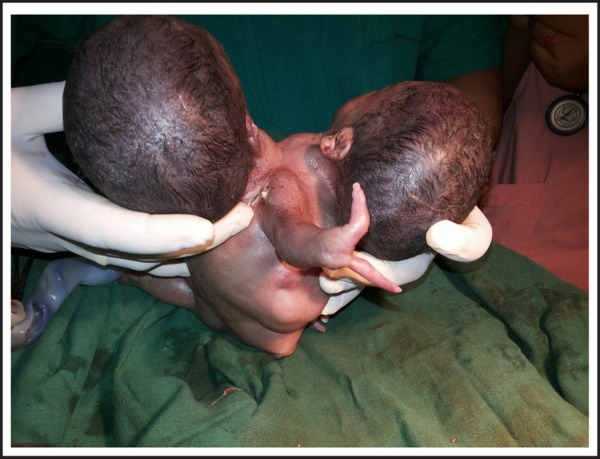
Conjoined Twins.

**Figure 2.**
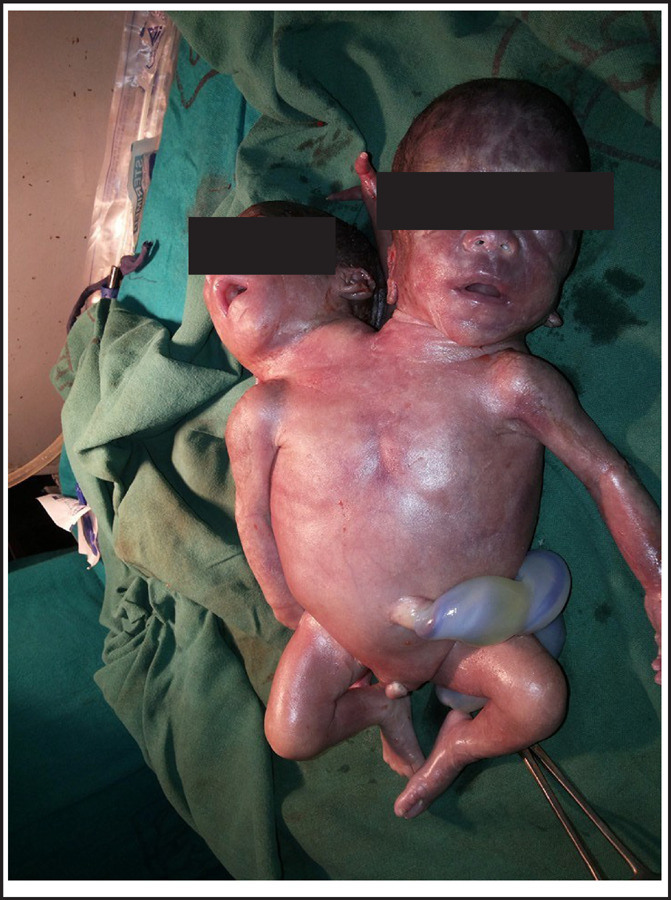
Conjoined Twins.

Fetal outcome:conjoined twin of Parapagusdicephalus type; both male; Apgar score- 1/10, 1/10;weight-2.0kg and time of birth- 2:20 PM (23^rd^ August 2015) (Figure 1,2).

Examination of conjoined twins showed:two heads with normal face; fused thorax; threelimbs each upper and lower; middle upper limb presenting with two fingers; no anal opening; heart rate: <100beats per minute and no spontaneous respiration.

With oxygen supplementation baby was planned to transfer to neonatal unit but patient party signed for do not resuscitate status with agreement for continued oxygen supplementation and baby expired on same day at one hour of life at operation theatre itself.

## DISCUSSION

Conjoined twins are classified according to the site of union^3^, ventral - Rostral, Caudal, Lateral and Dorsal.

The most common type is Thoracopagus (19%).^1–3,5,6^ It is identified using ultra-sonography at mid-pregnancy by careful examination of point of connection and organ (s) involved which even determines the viability and chances of successful separation following surgery. In late pregnancy, when there is diminished amniotic fluid and increased fetal crowding, Magnetic resonance imaging (MRI) will be the useful investigation.^1^ Termination of pregnancy is opted when heart or brain is shared as attempts to separate usually fail.^1,3^ Once diagnosis is made, plans should be made for caesarean delivery unless there is possibility of safe vaginal delivery. Vaginal delivery has risk of dystocia and; trauma to uterus and cervix.^1^ Outcome of conjoined twins are usually poor. About 40% will be stillborn and about 35% will die within one day of delivery.^3,5^ Only little hope of independent life is through surgical separation when there is absence of malformations, lack of bone unions and existence of separate heart and brain.^3^

Conjoined twins are more common in females but these in contrast were males. Among the conjoined twins, Thoracophagus is most common type^1–3^ but contrast to this in our case is Parapagusdicephalus type. Regular ante-natal check up and anomaly scanning at mid-trimester helps in early diagnosis^1^ so that timely discussion with parents regarding continuation or termination of pregnancy could be done and decision on mode of termination could be planned. Although it cannot be prevented but proper management can be done once diagnosis is made which is even aided by easy availability of MRI at times of diagnostic dilemma usually on later weeks of gestation and regarding sharing of vital organs.^1,3^

Though we call many cases to be rare but we may face them at any centre at any time so proper counseling to the patient and patient party should be provided regarding management plan, their pros and cons and their chances of recurrence in future pregnancies. Our plan of management should depend on condition of twins along with treatment options available and feasible at our centre or part of the globe considering the wish of parents as well.

**Consent: JNMA Case Report Form was signed by patient's father and the original is attached with the patient chart.**
